# Effects of hyperbaric oxygen therapy on human musculoskeletal pathologies in clinical studies: a systematic review

**DOI:** 10.1186/s12891-026-10254-9

**Published:** 2026-08-10

**Authors:** Luise Weinrich, Thomas Tischer, Ludwig Schlesiger, Milena L. Pachowsky, Lorenz Hoos

**Affiliations:** 1 Clinic for Orthopaedics and Trauma Surgery, Malteser Waldkrankenhaus St. Marien, Erlangen, Germany; 2 Department of Orthopaedics, University Medical Centre Rostock, Rostock, Germany; 3 DIAKO Ev. Diakonie-Krankenhaus gemeinnützige GmbH, Bremen, Germany; 4 Department of Medicine 3 – Rheumatology and Immunology and Deutsches Zentrum für Immuntherapie (DZI), Friedrich- Alexander-University Erlangen-Nürnberg and Universitätsklinikum Erlangen, Erlangen, Germany

**Keywords:** Hyperbaric oxygen therapy, Sports medicine, Muscle, Bone healing, Ligament healing

## Abstract

**Background:**

Hyperbaric oxygen therapy (HBOT) has gained increasing interest in regenerative medicine for its capacity to enhance tissue oxygenation, reduce inflammation, and accelerate healing. While its use in musculoskeletal medicine is expanding, particularly among athletes and in sports orthopaedics, the clinical evidence base remains fragmented across indications and has not been systematically evaluated.

**Purpose:**

The aim of this systematic review was to synthesize the available clinical evidence for HBOT in human musculoskeletal pathologies, with focus on its application in orthopaedic medicine.

**Methods:**

A systematic PubMed and LIVIVO search identified clinical studies (January 1996 - January 2026) investigating intervention-based studies using HBOT for musculoskeletal conditions in humans. Exclusion criteria were combinatory therapies, transplantations, radiation injuries, osteomyelitis, spine and oral surgery. Data on study design, HBOT protocols and their outcomes was collected. A qualitative synthesis was performed due to heterogeneity in study designs and outcome measures. Risk of bias was assessed using Cochrane RoB 2 tool (RCTs) and ROBINS-I V2 (non-RCTs). Studies were categorized by pathology (muscle/tendons/ligament injury; avascular necrosis (AVN) and bone marrow edema (BME)).

**Results:**

Nineteen studies involving 612 patients met our inclusion criteria. Conditions included exercise-induced muscle damage (EIMD) and its symptomatic manifestation delayed onset muscle soreness (DOMS), ligament injuries, AVN, BME, and tendon perfusion. Evidence for HBOT’s effects in DOMS/EIMD was limited, with RCTs showing no significant benefit. Conversely, AVN and BME studies reported consistent improvements in imaging, pain and functional scores, particularly with prolonged protocols. Limited evidence suggested potential benefits in ligament healing; tendon oxygenation effects were inconclusive. HBOT protocols ranged from 1 - 12 sessions for soft-tissue and 20 - 80 sessions for bone injuries, mainly using 60 to 90 minute sessions with 100% oxygen. Adverse events were rare and mild. Most RCTs showed low risk of bias; non-randomized studies had serious-to-critical bias due to confounding and measurement issues.

**Conclusions:**

HBOT may have a role as an adjunctive therapy for musculoskeletal pathologies, especially AVN and BME, where the most consistent evidence was observed, while its benefit in ligament injuries, muscle recovery, and tendon pathology remains inconclusive and requires higher-quality trials to substantiate.

**Clinical relevance:**

This topic is directly relevant to orthopaedic and sports medicine practitioners considering its off-label use. This review identifies conditions with the most consistent clinical benefit, highlights the limitations of current evidence, and defines priorities for future high-quality trials to guide safe and effective clinical implementation.

**Study design:**

Systematic review.

**Supplementary Information:**

The online version contains supplementary material available at 10.1186/s12891-026-10254-9.

## Background

Hyperbaric oxygen therapy (HBOT), the intermittent inhalation of 100% oxygen under pressures exceeding 1.3 atmosphere absolute (ATA), has been utilized for over a century in various medical disciplines [1, 2].

Initially rooted in diving and wound management, slowly but surely an expanding interest as an effective adjunct in musculoskeletal medicine, particularly in sports orthopedics and regenerative rehabilitation, has been happening due to its support in potential acceleration of healing and potential of oxygenation in underperfused tissue [3, 4]. In recent years, HBOT has gained traction among professional athletes, benefiting from its recovery-enhancing effects [5–9]. Athletes frequently suffer musculoskeletal injuries demanding rapid and complete recovery, intensifying the need for therapeutic strategies that accelerate tissue repair and enable safe return to play [10]. HBOT, with its capacity to increase tissue oxygenation and modulate inflammation, has been proposed as one such strategy, also among the physically active working society [9, 11].

HBOT’s primary mechanism, namely elevating oxygen tension in hypoxic tissue, has been shown to stimulate angiogenesis, reduce inflammation, and accelerate cellular repair, offering theoretical benefits in soft tissue and bone healing [12, 13]. Given this, it also exerts therapeutic effects through several mechanisms relevant to musculoskeletal healing. By elevating oxygen partial pressure in plasma, HBOT enhances oxygen delivery to hypoxic or inflamed tissues, even in areas with poor perfusion such as tendons, cartilage, or ischemic bone [12, 13]. This oxygen surplus has been shown to promote angiogenesis, fibroblast proliferation, and collagen synthesis - key processes in tendon and ligament repair [14–16]. Preclinical evidence has suggested potential HBOT effects across musculoskeletal tissues, though findings varied by tissue type and model [17]: In bone, HBOT seems to stimulate osteoblast activity, enhance mineralization, and upregulate pro-regenerative transcription factors like Runt-related transcription factor 2 (RUNX2) and Piezo1-YAP signaling [18]. In muscle, it may reduce secondary damage through modulation of oxidative stress and inflammatory pathways, while improving mitochondrial function and vascular remodeling [15, 19, 20].

Therefore, the aim of this systematic review is to synthesize the clinical evidence for application in common orthopedic problems. Given the increasing off-label and experimental use of HBOT in musculoskeletal rehabilitation [21], particularly in the athletic populations, this systematic review aims to consolidate existing evidence in human trials. It evaluates HBOT’s impact on clinical, imaging, and functional outcomes across various orthopedic conditions to better define its therapeutic role, efficacy, and translational potential within sports and orthopedic medicine.

## Methods

### Search strategy

**Fig. 1:**
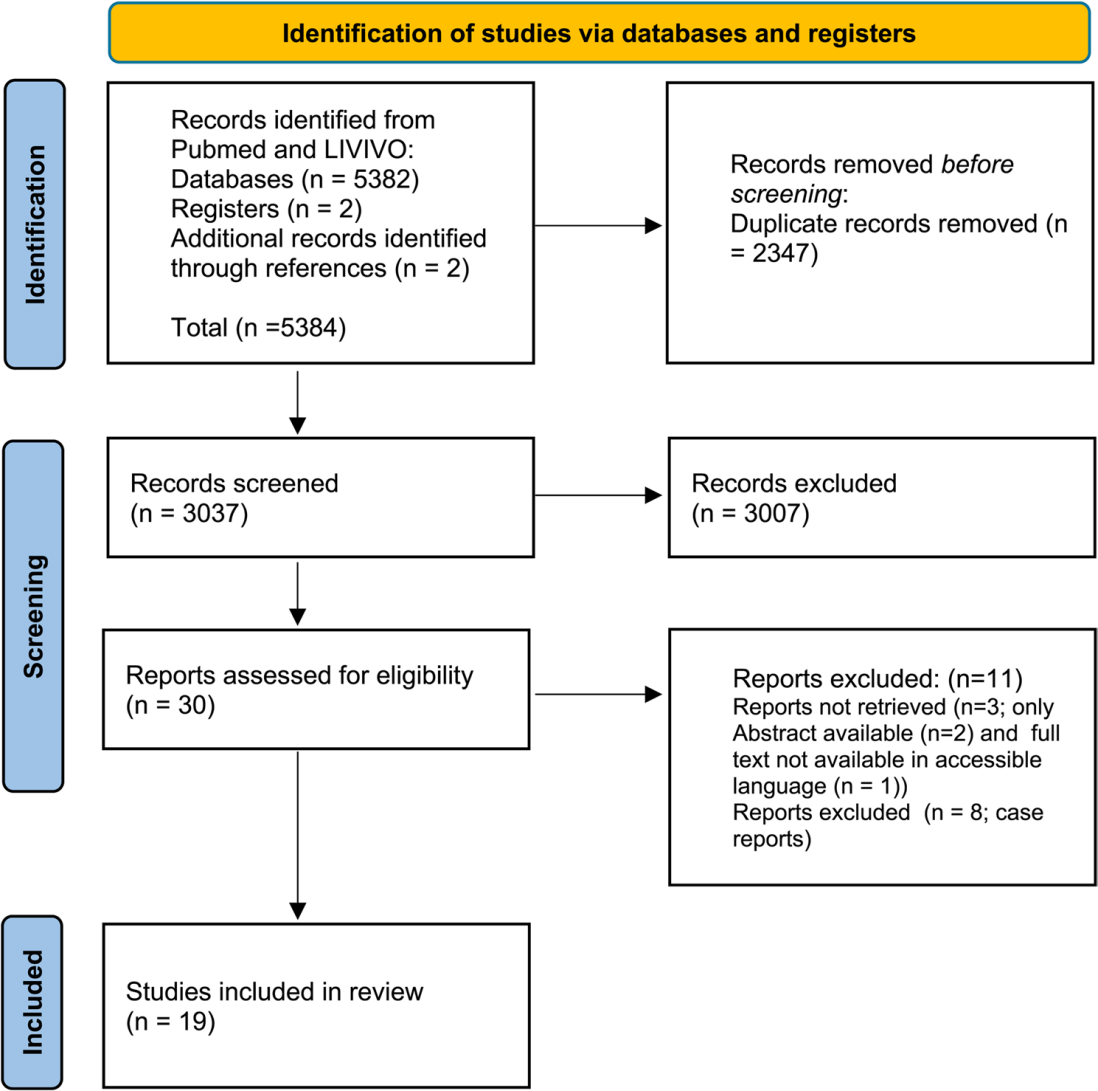
PRISMA flowchart of the study selection process [22]

A systematic literature search was performed between January 2026 on the electronic databases of PubMed and LIVIVO (operated by ZB MED – Leibniz Information Centre of Life Sciences; search engine for life sciences literature and information including medicine, health, nutritional and environmental sciences) according to the Preferred Reporting Items for Systematic Reviews and Meta-Analyses (PRISMA) guidelines (Fig. 1).

Clinical studies focusing on the effect of hyperbaric oxygen therapy (HBOT) on musculoskeletal tissues, namely bone, muscle, cartilage, tendon and ligament were included based on the following inclusion criteria: Clinical research articles published between January 1996 and January 2026, articles written in English or German, single treatment with HBOT to improve musculoskeletal pathologies or show effects of HBOT on those tissues. Exclusion criteria were: Non-clinical study regardless of type; systematic reviews and meta-analyses, congress abstracts, articles written in other languages than English or German, studies with non-singular use of HBOT / combination therapies (e.g. HBOT and Platelet-Rich Plasma (PRP)), studies involving transplantations, spinal or oral/maxillofacial surgery, infections or radiation therapy. The term ‘long covid’ was explicitly excluded to prevent retrieval of the large volume of HBOT literature pertaining to post-COVID rehabilitation, which falls outside the musculoskeletal scope of this review.

The following search strings were used for PubMed: *(“hyperbaric oxygen therapy” OR “hyperbaric oxygen” OR “hyperbaric oxygenation” OR HBO OR HBOT OR hyperoxygenation OR “mild hyperbaric oxygen”) AND (muscle OR ligament OR ACL OR tendon OR cartilage OR bone OR fracture OR “avascular necrosis” OR osteomyelitis OR sport OR sports OR recovery OR “sports medicine” OR athlete OR athletes) NOT (“long covid” OR “chronic wound” OR “carbon monoxide poisoning”) AND 1996:2024[dp]* and 2. for LIVIVO: (“hyperbaric oxygen therapy” OR “hyperbaric oxygen” OR “hyperbaric oxygenation” OR HBO OR HBOT OR hyperoxygenation OR “mild hyperbaric oxygen”) AND (muscle OR ligament OR ACL OR tendon OR cartilage OR bone OR fracture OR “avascular necrosis” OR osteomyelitis OR sport OR sports OR recovery OR “sports medicine” OR athlete OR athletes) AND PY="1996:2024” (including all Life Science databases). After deduplication this led to 3,037 titles. The flow-chart reported in Fig. 1 describes the systematic review process on 6 January 2026.

All potentially relevant titles and abstracts were then screened by two epidemiologically trained researchers (LW, TT) who excluded irrelevant articles, while a third author (LH) was involved to remove any discrepancies. Two reviewers (LW, LH) reviewed each study individually. The study design was categorized, and a level of evidence was assigned based on Oxford Centre for Evidence-Based Medicine (CEBM) recommendations. Disagreement was solved by consensus.

The following information was extracted: authors, title, year of publication, study type, level of evidence, number of patients, anatomic region, type of treatment/ intervention/ pathology, study concept, HBOT characteristics (chamber time in min, number of sessions, time period of sessions in days, oxygen level in %, amount of pressure in Atmosphere Absolute (ATA)), HBOT groups and control groups, main results and conclusions. Furthermore, each article was assigned to a corresponding subspecialty field. The following subspecialty fields were defined for that purpose: Exercise-Induced Muscle Damage or Injuries; Tendons and Ligaments Adaptation; Avascular Necrosis of the Femoral Head/Condyle and Bone Marrow Edema. A qualitative narrative synthesis was performed instead of a quantitative meta-analysis due to heterogeneity in study designs, HBOT protocols, and outcome measures. Studies were grouped by pathology category for synthesis.

The risk of bias of the included studies was assessed with two validated tools, depending on the study design. For randomized controlled trials (RCTs), the revised Cochrane Risk of Bias tool for randomized trials (RoB 2), which evaluates five domains, was applied. Each domain was rated as low risk, some concerns, or high risk [22]. For non-randomized studies, The Risk of Bias in Non-randomized Studies – of Interventions, Version 2 (ROBINS-I V2) assessment tool, which covers seven domains, was used. Each domain was rated as low risk, moderate risk, serious risk or critical risk [23]. Assessments were conducted independently by two reviewers, with disagreements resolved through discussion.

This review was not prospectively registered, and no formal review protocol was prepared prior to commencement.

## Results

### Study characteristics

This systematic review included 19 studies investigating the effects of hyperbaric oxygen therapy (HBOT) on musculoskeletal conditions in human subjects, selected from a total of 3035 published articles (Fig. 1). A total of 612 patients were included across all studies, with sample sizes ranging from 12 to 73 participants or patients. The studies addressed a range of clinical conditions, including exercise-induced muscle damage (EIMD), delayed onset muscle soreness (DOMS) – as symptomatic consequence, medial collateral ligament (MCL) injuries, ankle sprains, bone marrow edema (BME), and avascular necrosis (AVN) of the femoral head or condyle.

Patient demographics were inconsistently reported across studies. Age ranged widely, from pediatric populations in AVN studies to middle-aged and older adults in bone marrow edema cohorts, reflecting the heterogeneous clinical conditions investigated. Sex was explicitly reported in all studies with predominance of male athletes in muscle and ligament studies and the mixed sex distribution in bone studies.

Most studies were Level 2 or Level 3 evidence, with 7 randomized controlled trials (RCTs) and 12 cohort or comparative studies. HBOT protocols varied widely, with treatment pressures ranging from 1.3 to 2.5 ATA, session durations from 60 to 240 min, and total number of sessions ranging from 1 to over 130.

### Effects on exercise-induced muscle damage or injuries

Seven studies examined the effect of HBOT on muscle recovery following exercise-induced damage (EIMD) or its symptomatic manifestation, delayed onset of muscle soreness (DOMS) as well as acute soft tissue injury, mostly in athletes or healthy volunteers. The constructs of EIMD and DOMS are grouped together in this section as the included studies evaluated them as part of the same recovery process, often measuring both simultaneously. These studies employed eccentric muscle loading models, including isolated leg extension or sport-specific muscle trauma protocols. HBOT sessions ranged from 1 to 7 sessions, whereas 3–5 sessions were common (Table 1). The RCTs in this group were generally well-conducted, with most showing low risk of bias.

**Table 1:** Effects of hyperbaric oxygen therapy in exercise-induced muscle damage or injuries

Author	Title	Year	Study Type	LOE	*N*	anatomic region / study concept	HBOT Group	Control Groups	Results	Key Conslusions
Babul S et al. [24]	Effects of intermittent exposure to hyperbaric oxygen for the treatment of an acute soft tissue injury.	2003	RCT	2	16	Subjects performed 300 maximal voluntary eccentric contractions of nondominant leg at a slow speed on a dynamometer to elicit muscle damage and injury.	2.0 ATA / 60 min / 4 sessions / 100% Ox	1,2 ATA / 60 min / 4 / 21%	Analyses revealed no significant differences between groups for treatment effects for any of the dependent variables (pain, strength, quadriceps circumference, CK, malondialdehyde, or MRIs).	HBOT is not effective in the treatment of exercise-induced muscle injury as indicated by the markers evaluated.
Webster AL et al. [25]	Effects of hyperbaric oxygen on recovery from exercise-induced muscle damage in humans.	2002	RCT	2	12	Strenuous eccentric exercise protocol designed to elicit muscle damage within M. gastrocnemius, received either HBOT or mild HBOT afterwards	2,5 ATA / 60 min / 3 sessions / 100% Ox	1.3 ATA / 60 min / 3 / 21%	There was little evidence of a difference in recovery rate between the HBOT and CG. Faster recovery was observed in the HBOT group only for isometric peak torque and pain sensation and unpleasantness.	HBOT cannot be recommended as an effective method of treatment of this form of muscle injury.
Harrison BC et al. [26]	Treatment of exercise-induced muscle injury via hyperbaric oxygen therapy.	2001	Non-randomized-controlled cohort study	3	21	6 sets of eccentric repetitions with load equivalent to 120% of concentric maximum were performed; forearm flexor CSA and T2 relaxation time via MRI, isometric strength and rating of perceived soreness of the forearm flexors and serum CK	2,5 ATA / 100 min / 5 sessions / 100% Ox	1 CG without HBOT; 1 group with delayed HBOT: 2,5 ATA / 100 min / 4 / 100%	Baseline CSA values were similar across groups. All groups showed significant CSA increases with no group differences. T2 relaxation times also increased significantly without differences between groups. Isometric strength, serum CK levels, and perceived soreness confirmed muscle injury in all groups, with no signifcant differences.	HBOT was not effective in the treatment of exercise-induced muscle injury as indicated by the markers evaluated.
Mekjavic IB et al. [27]	Hyperbaric oxygen therapy does not affect recovery from delayed onset muscle soreness.	2000	RCT	2	24	Test maximal isometric strength (preexercise) of elbow flexors after high-force eccentric workout of the elbow flexor muscle group to induce DOMS; measurements: max. isometric muscle strength of elbow flexor muscles, upper arm circumference, transcutaneous pO2 and rating muscle soreness.	2,5 ATA / 60 min / 7 sessions / 100% Ox	2,5 ATA / 60 min / 7 / 8%	Isometric strength decreased significantly from preexercise levels to postexercise levels for the HBOT group for the placebo group. Over the 10-d recovery period, there was no difference in the rate of recovery of muscle strength between the two groups. Perceived soreness peaked at about 48 h after exercise with no difference between groups. Also, the exercise-induced increases in arm circumference were similar in the two groups.	HBOT is not an effective therapy for the treatment of DOMS.
Staples JR et al. [28]	Effects of hyperbaric oxygen on a human model of injury.	1999	RCT	2	66	AR: muscle / SC: untrained subjects underwent muscle soreness induction and were then treated with HBOT; recovery was measured using a leg dynamometer to assess eccentric torque and visual analog pain scales was used to track pain perception	2.0 ATA / 60 min / 5 sessions / 100% Ox; (treatments at 0, 24, 48 h; sham at 72, 96 h)	Delayed HBOT G: sham at 0, 24 h; then HBOT at 48, 72, 96 h sham G: 1.2 ATA / 60 min / 5 / 21%; CG: no treatment	HBOT enhanced quadriceps torque recovery after DOMS but did not significantly reduce pain. Phase 1 showed greater eccentric torque recovery in the HBOT group, and Phase 2 confirmed superior recovery after 5 days of HBOT compared to sham treatment.	HBOT enhances the recovery of muscle strength following DOMS but does not significantly reduce pain perception. Findings suggest that HBOT may be a useful tool for improving muscle function recovery after EIMD, though effects on pain relief remain inconclusive.
Woo J et al. [29]	Effects of Hyperbaric Oxygen Therapy on Inflammation, Oxidative/Antioxidant Balance, and Muscle Damage after Acute Exercise in Normobaric, Normoxic and Hypobaric, Hypoxic Environments: A Pilot Study.	2020	Pilot RCT	2	18	Participants exercised, divided randomly to Gs. G1: HBOT after exercising in normobaric, normoxic conditions (= HNN), G2: HBOT after exercising in hypobaric, hypoxic conditions (= HHH). Blood samples measured inflammation, oxidative stress, and muscle damage.	2.5 ATA / 60 min / 1 session / 100% Ox	1 CG without HBOT	Exercise significantly increased markers of inflammation and muscle damage (fibrinogen, IL-6, CK, LDH) across all groups. HBOT effectively reduced these markers, particularly in the HNN and HHH groups, though some markers remained elevated compared to baseline.	Exercise induced inflammation and muscle damage, regardless of environment. HBOT effectively reduced these effects during recovery.

*Abbreviations*: *AR*, anatomic region, *ATA* Atmosphere Absolute, *CG* control group, *CK* creatine kinase, *CSA* cross-sectional area, *DOMS* delayed onset muscle soreness, *EIMD* exercise-induced muscle damage, *G* group, *HBOT* Hyperbaric oxygen therapy, *IL-6* interleukin-6, *LDH* lactate dehydrogenase, *LOE* Level of evidence, *M.* muscle, *MCL* medial collateral ligament, *max.* maximum, *MRI* magnetic resonance imaging, *N* number, *NIRS* near-infrared spectroscopy, *Ox* Oxygen, *d* day, *RCT* randomized controlled trial, *SC* study concept, *St02* oxygen saturation, *THb* blood volume, *VAS* visual analogue scale

Significant improvements were reported markers of inflammation and muscle damage in one pilot RCT [29], suggesting a potential anti-inflammatory effect of HBOT post-exercise.

But no significant benefits of HBOT were found in most RCTs [25–28, 30]. Pain, creatine kinase levels (CK), and recovery of muscle strength were largely unaffected by HBOT compared to controls. Kubo et al. showed no improved muscle perfusion using near infrared spectroscopy (NIRS) [31].

### Effects on tendons and ligaments

The above mentioned study by Kubo et al., that investigated acute and chronic circulatory effects of HBOT on tendons and muscles using NIRS, reported improved tendon perfusion and oxygenation especially after the longer experiment, using 12 sessions over 6 weeks with only 50% oxygen saturation [31]. The studies in this group were methodologically heterogeneous with two non-randomized studies carrying serious risk of bias.

Yagishita et al. observed faster return-to-play times and less pain in Japanese rugby athletes with medial collateral ligament (MCL) injury, suggesting clinical benefits in ligament injuries after only 3–5 sessions though carrying serious risk of bias [32].

Similarly, Abbadi et al. reported complete pain and swelling resolution after seven HBOT sessions at 2.5 Atmosphere Absolute (ATA) in acute ankle sprains, indicating potential for accelerated ligament healing [33]. However, Borromeo et al. found no significant benefit of HBOT over air in functional recovery or return-to-play time in a double-blind trial on ankle sprains [34] (Table 1).

### Effects on avascular necrosis of the femoral head and condyle

Nine studies evaluated the effect of HBOT on AVN of the femoral head or condyle, including both early-stage lesions (Stage I/II) and chronic non-traumatic necrosis.

Retrospective cohorts consistently reported that HBOT stabilized or lessened the amount of edema in magnetic resonance imaging (MRI), reduced the need for joint replacement, and led to good patient-reported outcomes [35–40]. Clinical scores (Oxford Hip/Knee Score, Short Form 12 Dimensions (SF-12), Western Ontario and McMaster Universities Arthritis Index (WOMAC)-Score, modified Harris Hip Score) improved in most cases [35, 38, 41, 42]. Treatment protocols were typically long, with 25 to 135 sessions, highlighting the chronic nature of AVN and the potential requirement for extended HBOT protocols (Table 2). It should be noted that most AVN studies were retrospective observational cohorts, Chandrinou et al. Being a prospective observational study, rated as having serious to critical risk of bias, primarily due to confounding and absence of control groups.

**Table 2:** Effects of hyperbaric oxygen therapy in tendons, ligaments and muscles

Author	Title	Year	Study Type	LOE	*N*	anatomic region / study concept	HBOT Data	Control Groups	Results	Key Conslusions
Kubo K et al. [31]	Acute and chronic effects of hyperbaric oxygen therapy on blood circulation of human muscle and tendon in vivo.	2012	Non-randomized intervention-study	3	12	AR: tendon and muscle / SC: investigation on how HBOT acutely and chronically affects blood circulation (blood volume and oxygen saturation) in human muscle and tendon in vivo, using NIRS for muscle and red laser light technology for tendon.	1.3 ATA / 60 min / 1 or 12 sessions/ 50% Ox	no CG	Oxygen saturation of the tendon increased during HBOT while muscle oxygenation remained unchanged. After six weeks, resting blood volume and tendon oxygen saturation decreased, but muscle values did not. In experiment 1, THb of the muscle increased, but StO2 remained stable, whereas both THb and StO2 of the tendon increased. In experiment 2, the response patterns were similar across sessions. After six weeks, resting THb and StO2 of the tendon were lower, but muscle values remained unchanged.	Oxygen saturation of the tendon increased during hyperbaric oxygen therapy, while muscle oxygenation remained unchanged. After six weeks, resting tendon oxygen saturation decreased. Lower pressure and oxygen density may be sufficient for effective therapy.
Yagishita K et al. [32]	Effects of hyperbaric oxygen therapy on recovery acceleration in Japanese professional or semi-professional rugby players with grade 2 MCL injury of the knee: A comparative non-randomized study.	2019	Comparative non-randomized study	3	32	Rugby players with grade 2 MCL injuries were studied. In the HBOT group therapy began immediately. Same-day VAS scores were compared before and after HBOT. Return-to-play time was retrospectively evaluated between HBOT and non-HBOT groups.	2.8 ATA / 60 min / 4.6 ± 0.7 (3–5) sessions / 100% Ox	1 CG without HBOT	VAS scores for walking and jogging pain improved significantly after HBOT (*p* < 0.001). Return-to-play time was shorter in the HBOT group (31.4 ± 12.2 days) than in the non-HBOT group (42.1 ± 15.8 days, *p* < 0.05). Early HBOT (≤ 2 days) led to faster recovery (27.9 ± 9.3 days) than delayed HBOT (39.0 ± 13.1 days), but the difference was not significant.	HBOT may reduce pain and accelerate the return to play in athletes with grade 2 MCL injury of the knee in this non-randomized study.
Abbadi et al. [33]	The role of Hyperbaric Oxygen in managing ankle sprains	2003	RCT	2	36	Patients with acute ankle sprains, devided into 3 groups, then different HBOT pressures over 5 days.	G2: 2 ATA, G3: 2.5 ATA / 90 min / 7 sessions / 100% Ox	1 CG (G1) with HBOT at 1 ATA	Pain and swelling improved significantly in patients treated with HBOT at 2.5 ATA, with complete resolution by final session. Edema reduction was greater with 2.5 (97%) than 2.0 (80%) and 1.0 ATA (30%). No significant improvement was observed in the CG. Pain relief was reported from first session in both HBOT groups, with complete pain resolution by third session at 2.0 ATA.	Hyperbaric oxygen therapy is the best choice for the rapid recovery of acute ankle sprain.
Borromeo et al. [34]	Hyperbaric Oxygen Therapy For Acute Ankle Sprains	1997	Randomized double-blinded Controlled Trial	2	32	Patients with acute lateral ankle sprains were randomized within 72 h to HBOT or CG. Both groups received three chamber sessions over 7 days. Ankle function, pain, edema, and return-to-play time were assessed.	2.0 ATA / 90 and 60 min / 3 sessions / 100% Ox	1 CG (G1) with HBOT at 1.1 ATA	Ankle function improved in both groups, with a greater change in the HBOT group (Δ = 5.9 ± 0.4) than CG (Δ = 4.5 ± 0.5, *p* < 0.05). Final function scores were not significantly different. Pain and edema decreased similarly in both groups, with no significant differences. Range of motion improved equally. Time to full recovery was comparable (HBOT: 16.0 ± 6.3 days; control: 15.4 ± 2.8 days). Regression analysis identified initial injury severity and age, but not HBOT, as significant predictors of recovery time. Recovery occurred within 15–16 days in both.	HBOT did not improve recovery outcomes compared to air exposure in acute ankle sprains. Both groups regained full function within 15–16 days, with age and injury severity being the only predictors of recovery time.

Kubo et al. (2012) [31] is included in this table rather than Table 1 as the study simultaneously examines HBOT effects on both tendon and muscle blood circulation, reflecting its thematic overlap with the connective tissue studies in this group

*Abbreviations*: *AR* anatomic region, *ATA* Atmosphere Absolute, *CG* control group, *G* group, *HBOT* Hyperbaric oxygen therapy, *LOE* Level of evidence, *MCL* medial collateral ligament, *N* number, *NIRS* near-infrared spectroscopy, *Ox* Oxygen, *RCT* randomized controlled trial, *SC* study concept, *St02* oxygen saturation, *THb* blood volume, *VAS* visual analogue scale

One cohort study showed a comparable outcome of HBOT to core decompression [37].

With an eye on pediatric cases, Chandrinou et al. and Scherer et al. provided observational support for HBOT in AVN, showing improved quality of life and radiographic stabilization in the younger population, too [42, 43].

The Currie et al. study provided long-term observational data over 30 years, though the specific HBOT protocol and control group details were not reported in sufficient detail; this study was retained as it contributed relevant long-term outcome data in this narrative synthesis context (Table 3).

**Table 3:** Effects of hyperbaric oxygen therapy in avascular necrosis and bone marrow edema

Author	Title	Year	Study Type	LOE	*N*	anatomic region / study concept	HBOT Group	Control Groups	Results	Key Conslusions
Scherer A et al. [43]	Evaluation of Aseptic Osteonecrosis in Children over the Course of Hyperbaric Oxygen Therapy.	2000	Retrospective Controlled Cohort Study	3	20	Evaluation of effects of HBOT on AVN in children undergoing chemotherapy using MRI; retrospective analysis of 72 MRI, G1: receiving only relief of weightbearing structures, G2: additional HBOT. Point-score system to assess MRI findings over time.	2,5 ATA / 90 min / 30–60 (average 43) sessions / 100% Ox	CG only conservative treatment (weightbearing)	G2 initially showed more severe findings (average score: 3.4) in comparison to G1 (average score: 2.65). During the follow-up time period the average scores rose to 3.2 score-points in G1 and 4.1 points in G2. No statistically significant difference was evident between the two groups in the course of AON.	No statistically significant improvement in MRI morphology with HBOT compared to weightbearing relief alone. However, HBOT appeared to help with pain alleviation, showed potential for treating early-stage AON.
Bosco G et al. [39]	Femoral condylar necrosis: treatment with hyperbaric oxygen therapy.	2018	Retrospective Cohort Study (observational)	4	37	Subjects with osteonecrosis of the knee were evaluated receiving HBOT; MRIs were taken before, within a year after HBOT, and in 14 patients, 7 years later. OKS were recorded.	2,5 ATA / 60 min / 67.9 ± 15 sessions / 100% Ox	non	After 30 HBOT sessions, 86% of patients improved in OKS, 11% worsened, 3% remained unchanged; all improved after 50 sessions. MRI after 1 year showed edema resolution in all but one patient. Pain, mobility, radiographic findings improved, with Aglietti stage decreasing from 1.7 ± 0.7 to 0.3 ± 0.6 (*p* < 0.01).	HBOT is effective for treating osteonecrosis of the knee. In this study, 95.4% improved after the first cycle, and all improved after the second. Further research is recommended.
Koren L et al. [38]	Hyperbaric oxygen for stage I and II femoral head osteonecrosis.	2015	Retrospective Cohort Study (observational)	4	68	Subjects with Steinberg stage I-II AVNFH, treated with HBOT were reviewed. MRI findings, joint survival via follow-up interviews, changes in mHHS and SF-12.	2-2.4 ATA / 90 min / 78.3 ± 24.2 sessions / 100% Ox	non	74 joints had pre- and posttreatment MRIs, 88% showing improvement. At an 11.1 ± 5.1-year follow-up, 93% of 58 joints survived. Mean mHHS rose from 21 to 81, and SF-12 physical and mental scores improved significantly. Stage I had 95% improvement, stage II 81%, idiopathic cases 93%, trauma cases 85%, and secondary cases 75%.	HBOT is effective in preserving the hip joint in stage I and II osteonecrosis of the femoral head.
Chandrinou A et al. [42]	Avascular necrosis of the femoral head: Evaluation of hyperbaric oxygen therapy and quality of life.	2020	Prospective Observational Study	3	73	Examination of HBOT effects on patients with early-stage AVNFH. Quality of life, hip function were assessed around HBOT and over five-month follow-up period.	2.0 ATA / 120 min / 20 sessions / 100% Ox	non	This study found that HBOT significantly improved joint pain and function in patients with early-stage avascular necrosis of the femoral head. Improvements observed across after 2 HBOT rounds.	HBOT does not induce alteration of quality of life and is well tolerated and accepted by patients.
Moghamis I et al. [37]	The outcome of hyperbaric oxygen therapy versus core decompression in the non-traumatic avascular necrosis of the femoral head: Retrospective Cohort Study.	2021	Retrospective Cohort Study	4	19	Patients with non-traumatic AVNFH were analyzed, evaluating the outcomes of HBOT and CD using hip scores and imaging over a minimum 12-month follow-up.	2.2 ATA / 90 min / 25–40 sessions / 100% Ox	Operative treatment: CD group without HBOT	This study compared CD and HBOT for stage II avascular necrosis of the femoral head. Both treatments showed significant improvement in hip function, with no significant difference between the two groups. Radiological progression occurred in some patients, but was not statistically different between CD and HBOT.	HBOT is as effective as CD for early-stage avascular necrosis of the femoral head, offering a non-invasive treatment option.
Currie JR et al. [36]	The use of hyperbaric oxygen for avascular necrosis of the femoral head and femoral condyle: a single centre’s experience over 30 years.	2024	Retrospective Study	4	14	Long-term outcomes of HBOT for AVNFH, focusing on radiological improvement, subjective benefits, joint replacement needs, and complications.	unknown	unkonwn	HBOT showed positive outcomes in both patient groups, with improved MRI scans and subjective reports. While some femoral head patients required surgery, all femoral condyle patients avoided it. Mild barotrauma was observed in few patients.	HBOT can prevent disease progression in femoral AVN, supporting its potential as a significant treatment option.
Ozturan B et al. [35]	Effectiveness of hyperbaric oxygen therapy in bone marrow edemas of the knee: A retrospective study.	2023	Retrospective Cohort Study	3	43	HBOT effect in addition to standard treatment (aspirin and bisphosphonates) on BME in patients without other joint conditions was investigated.	2.5 ATA / unknown / 20 sessions / 100% Ox	1 CG without HBOT	HBOT significantly reduced BME lesion size in the knee compared to standard treatment alone. The HBOT group showed a 35% reduction, while the non-HBOT group only showed a 5% reduction.	HBOT effectively reduces BME in the knee with no significant side effects, demonstrating faster healing compared to standard treatment.
Capone A et al. [41]	Hyperbaric oxygen therapy for transient bone marrow oedema syndrome of the hip.	2011	RCT	2	44	BME syndrome of the femoral head were compared; G1: pharmacological and HBOT, G2: pharmacological therapy alone.	2.2 ATA / 90 min / 40 sessions / 100% Ox	CG (G2) treated with protective weight-bearing and analgesics or NSAID	The overall average WOMAC score at 3 months was significantly higher (*p* < 0.001) for the HBOT group (70.8 points) compared with the CG (56.4 points). MRI at 3 months showed resolution of bone marrow oedema in 55.0% of the patients treated with HBOT compared with 28% in the CG.	HBOT appears to be effective in treating transient bone marrow oedema syndrome, resulting in an accelerated recovery of hip function compared to pharmacological therapy alone.
Etli I et al. [40]	Is Hyperbaric Oxygen Therapy Effective in Early-Stage Avascular Necrosis of The Femoral Head?	2021	Retrospective Cohort Study	4	25	HBOT effect in the treatment for early-stage avascular necrosis of the femoral head. Focus on clinical and radiological findings before and after treatment.	2.5 ATA / 90 min / 27–47 sessions / 100% Ox	non	After HBOT, patients experienced significant pain relief and functional improvement, with higher Harris Hip Scores and improved hip motion. However, radiological progression occurred in many patients, indicating that clinical benefits were not consistently matched by imaging findings.	HBOT led to significant improvements in pain, hip function, and range of motion, reflected by a marked increase in Harris Hip Scores. Despite the clinical benefits, radiological outcomes were variable, with disease progression observed in a substantial portion of patients.

*Abbreviations*: *AON* aseptic osteonecrosis, *ATA* Atmosphere Absolute, *AVN* avascular necrosis, *AVNFH* Avascular Necrosis of Femoral Head, *BME* bone marrow edema, *CD* core decompression, *CG* control group, *G* group, *HBOT* Hyperbaric oxygen therapy, *LOE* Level of evidence, *mHHS* Modified Harris Hip Score, *MRI* magnetic resonance imaging, *N* number, *NSAID* non steroidal anti-inflammatory drugs, *OKS* Oxford Knee Score, *Ox* Oxygen, *RCT* randomized controlled trial, *SF-12* Short Form 12 Dimensions, *WOMAC* Western Ontario and McMaster Universities Arthritis Index

In total, these results suggest that HBOT may delay disease progression in AVN, especially when applied in early stages, and could represent a viable non-surgical treatment option.

### Effects on bone marrow edema

Two studies assessed the role of HBOT in BME of the hip and knee using 20 and 40 HBOT sessions [35, 41] (Table 2). Both BME studies carried serious risk of bias.

Both studies reported significant radiological improvement in edema volume and pain reduction, particularly when HBOT was added to standard therapy like bisphosphonates. The session duration for Ozturan et al. was not explicitly reported in the original publication and could not be retrieved [35].

MRI-based endpoints showed volumetric improvement after 30–40 sessions of 2.5 ATA treatments (Table 2).

These findings support the use of HBOT as an adjunctive therapy for BME, particularly in patients with persistent pain or failed conservative therapy.

### Adverse effects

No study reported serious adverse events, though mild middle ear or sinus barotrauma was noted in 3 patients [36].

### Risk of bias

**Fig. 2:**
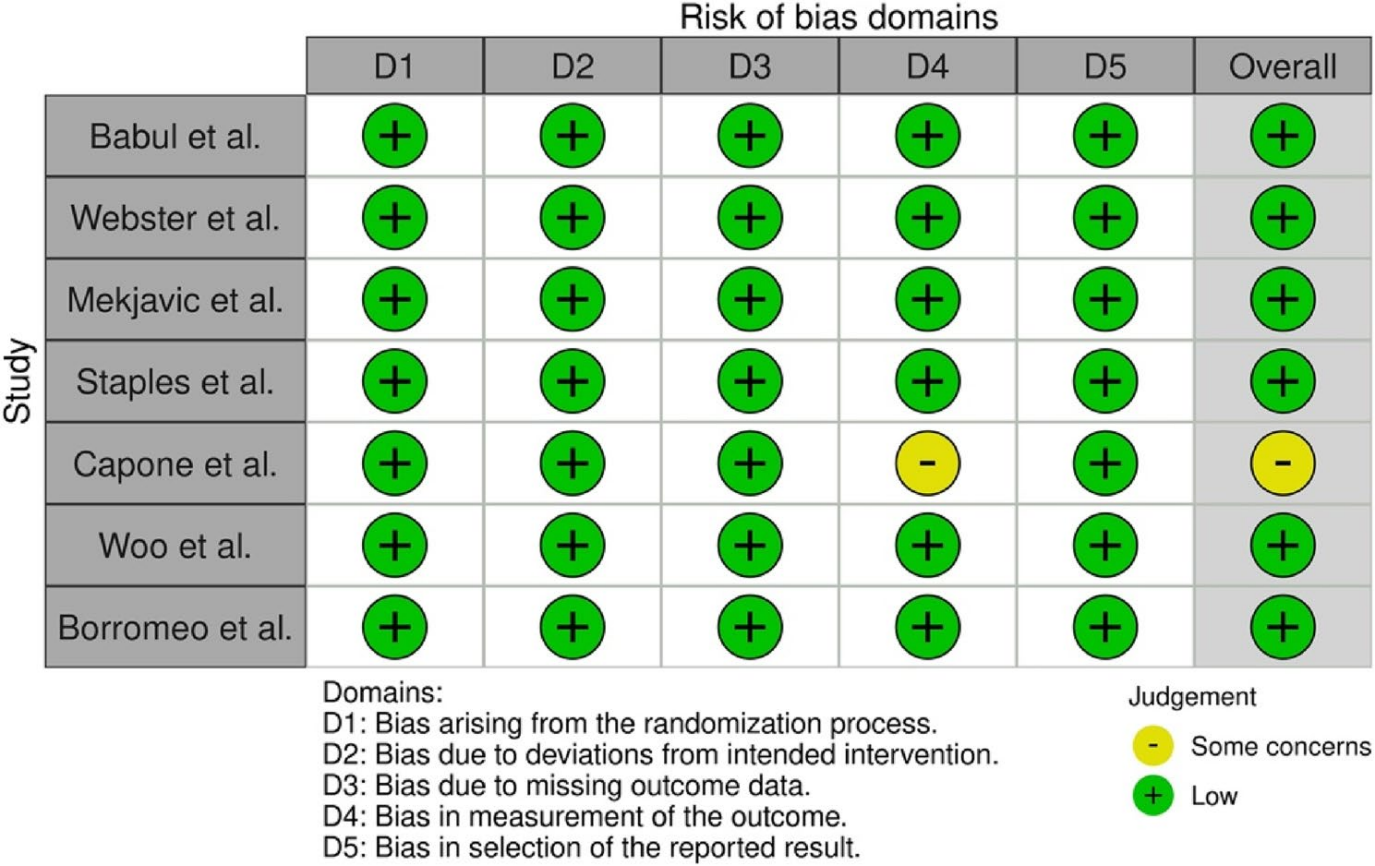
Risk of bias assessment for RCTs using RoB 2 tool [44]

**Fig. 3:**
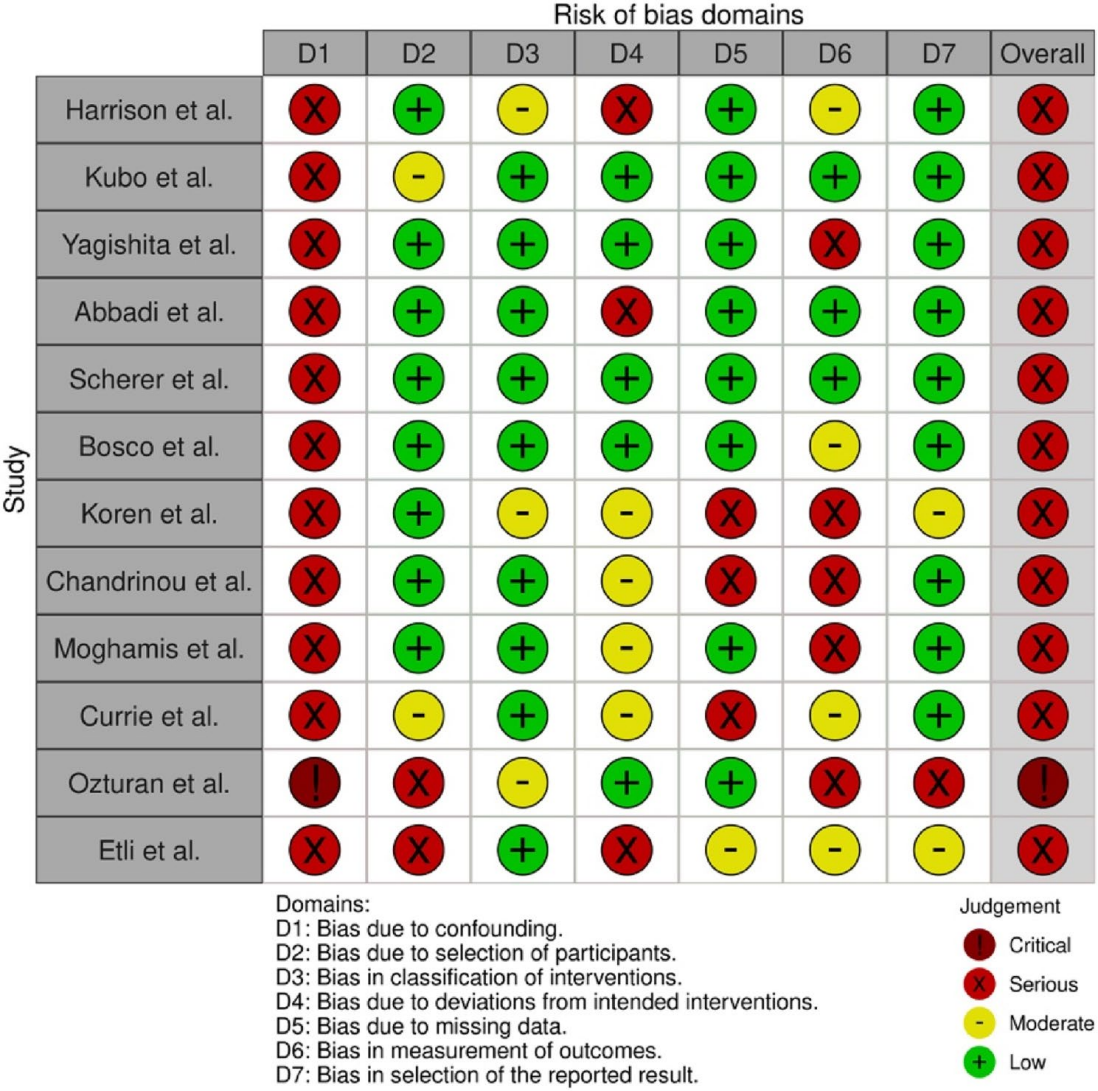
Risk of bias assessment for non-randomized studies using ROBINS-I V2 tool [44]

Among the seven RCTs assessed with the revised Cochrane Risk of Bias tool for randomized trials (RoB 2), six studies were judged to have a low overall risk of bias. One study, Capone et al., was assessed as having some concerns [41], primarily due to uncertainties in the selection of reported results (Figs. 2 and 3).

Twelve non-randomized studies assessed with the Risk of Bias in Non-randomized Studies - of Interventions, Version 2 assessment tool (ROBINS-I V2) were found to have a serious risk of bias, mainly due to confounding and measurement issues. One study, Ozturan et al., was rated as having a critical risk of bias [35], and was considered at high risk of yielding unreliable conclusions.

## Discussion

The results of this study show, that HBOT was used across a spectrum of conditions ranging from acute soft tissue injuries and ligament sprains to chronic degenerative bone disorders. Thereby, HBOT shows the most consistent clinical benefit in bony pathologies such as AVN and BME, where prolonged treatment protocols appear to improve radiological outcome measures, pain and functional outcomes. By contrast, evidence for its effectiveness in exercise-induced muscle damage (EIMD) remains limited and inconclusive, with most randomized controlled trials reporting no significant advantage over controls. Potential benefits were observed in ligament healing, particularly with short-term protocols, while findings regarding tendon perfusion were variable.

This systematic review is, to our knowledge, the first to comprehensively evaluate the effects of HBOT on musculoskeletal injuries in human subjects. Across 19 studies, our analysis revealed mixed but promising evidence. While certain clinical conditions benefit from HBOT, the overall clinical utility remains inconsistent, especially when contrasted with the robust and uniform findings from basic science.

Preclinical evidence suggests a potential role for HBOT in musculoskeletal healing, though findings varied by tissue type, injury model, and methodological quality [17]. Across these preclinical and mechanistic studies, reported improvements spanned angiogenesis and vascular remodeling, collagen synthesis and extracellular matrix organization, satellite cell activation and muscle regeneration, bone remodeling and mineralization, and modulation of inflammatory pathways, though the magnitude and consistency of these effects varied by tissue type and experimental model [45–49]. Some preclinical evidence suggested potential benefits in muscle regeneration following contusion injury [15, 49], extracellular matrix remodeling and neovascularization in ligament and tendon models [50, 51], and enhanced bone formation and mineralization in fracture and distraction osteogenesis models [47, 52, 53]. Cartilage repair, traditionally considered challenging, showed accelerated early healing with HBOT in two studies [54, 55]. These findings provide a biological rationale for investigating clinical applications in acute muscle injuries, ligament reconstruction, tendinopathy, bone fractures, avascular necrosis, and early osteochondral lesions, as often seen in sports orthopedic cases. However, clinical translation of these findings remains variable. The biological mechanisms have not yet translated into uniform clinical benefit across all musculoskeletal indications.

One likely reason is the lack of clearly defined clinical indications and standardized HBOT protocols. While HBOT is well-established in indications such as diabetic wound healing and decompression sickness [56], its application in musculoskeletal medicine remains off-label and inconsistently applied [21]. In this review, HBOT was used for a wide range of indications, including muscle soreness, ligament injuries, and bone conditions like AVN and BME. Treatment protocols varied substantially, with session counts ranging from a single exposure to over 130 sessions, pressures between 1.3 and 2.5 Atmosphere Absolute (ATA), and durations of 60 to 120 min (Tables 1 and 2). While short-term protocols were frequently used in athletic recovery and ligament injuries, chronic bone conditions typically used more prolonged exposure times. This variability complicates comparisons and underscores the need for consensus on optimal dosing, timing, and patient selection to maximize clinical benefits. The lack of standardization in the clinical trials might partly contribute to the inconsistent outcomes observed across different indications.

A specific example is the limited efficacy seen in studies on exercise-induced muscle damage (EIMD). Notably, these findings in muscle recovery come predominantly from RCTs with lower risk of bias, lending them comparatively more reliability than the positive signals observed in observational bone studies. On the other hand, the included trials administered only 1 to 5 HBOT sessions, typically ranging between 60 and 90 min (Table 1), which is far below the clinical standard of 20 to 40 sessions required to achieve lasting sustained tissue oxygenation effects [57, 58]. In contrast, studies investigating HBOT in AVN or BME used more extended protocols, demonstrating significant improvements in imaging and clinical outcomes (Table 2). However, as all AVN and BME studies were retrospective observational cohorts with serious to critical risk of bias, these findings should be interpreted as hypothesis-generating rather than conclusive. This suggests that short-term HBOT might be insufficient to trigger regenerative pathways in musculoskeletal tissues, particularly in muscle, which may require prolonged exposure to hyperoxia to activate satellite cells or modulate inflammation effectively [17]. On the other hand, HBOT plays a major role in hypoxic tissue areas, which generally does not include muscle as a well-vascularized tissue [48]. This variability also reflects in the two studies on ankle sprains. While Abbadi et al. reported complete resolution of pain and swelling after seven HBOT sessions at 2.5 ATA in acute ankle sprains [33], Borromeo et al. found no significant functional improvement compared to control when using only three sessions [34]. These findings emphasize how dosage and timing may critically influence clinical outcomes.

Mechanistically, HBOT enhances oxygen delivery even in poorly perfused tissues such as cartilage and tendons [12, 13, 50, 59, 60]. Animal studies suggest that this can stimulate collagen synthesis, angiogenesis, and extracellular matrix remodeling [51, 54], effects that may help mitigate degenerative changes and delay the onset of osteoarthritis. Despite its massive socio-economic burden and high prevalence in aging athletic and working populations, only one clinical study in our review addressed early osteoarthritic changes through the treatment of bone marrow edema, suggesting HBOT may play a role in disease modification when administered early [35].

This potential mechanism is supported by preclinical data: it has been shown to inhibit the High mobility group box 1 (HMGB1) / receptor for advanced glycation end products (RAGE) signaling pathway by upregulating miR-107 in osteoarthritic chondrocytes, thereby reducing inflammation and cartilage degradation [61]. Additionally, Wang et al. demonstrated that HBOT suppressed H-type angiogenesis in the subchondral bone by enhancing Prolyl Hydroxylase Domain Protein 2 (PHD2) expression and downregulating Hypoxia-inducible factor (HIF)-1α and vascular endothelial growth factor A (VEGFA), a mechanism linked to osteoarthritis progression [53].

As the interest in HBOT grows, so too does the need for structural integration in clinical settings. A registry system for HBOT, as proposed by Harlan et al., may support standardized data collection, patient selection, and protocol refinement [57]. Moreover, technological innovations such as portable HBOT chambers, already in use for emergency conditions like carbon monoxide (CO) poisoning, could expand access to outpatient or sports field applications in the future [62].

In modern sports medicine, optimizing recovery is more critical than ever [6]. In recent years, HBOT has gained traction among professional athletes, benefiting from its recovery-enhancing effects, Cristiano Ronaldo being one of the most widely cited examples [6–8, 32, 63–66]. Further these demands extend beyond elite performance in today’s working society. The use for accelerated recovery after musculoskeletal injury may be relevant across all physically active populations and their workplace as well [11, 67, 68].

Combination therapies also offer exciting prospects. Though excluded from this narrative synthesis, preclinical models have shown synergistic effects of HBOT with platelet-rich plasma (PRP) and bone marrow concentrate on osteochondral and bone repair [69–71]. Anti-inflammatory co-treatments such as ozone and HBOT have also shown promise in soft tissue injury models [72, 73]. In surgical settings in animal models, HBOT has been shown to support graft maturation and tendon-to-bone healing in anterior cruciate ligament (ACL) reconstruction models [60, 74, 75], and this principle could be transferred to post-operative protocols in human athletes.

Additionally given the high socioeconomic burden of osteoarthritis [76] and the relatively favorable cost profile of HBOT, its potential as a preventive or modifying therapy merits further investigation [77]. Given HBOT’s cost-effectiveness and minimal side effects [78], this warrants further investigation in orthopedic preventive care.

The methodological quality of the included studies was heterogenous. The randomized controlled trials (RCTs) were generally well-conducted, with six of seven showing a low risk of bias according to the revised Cochrane Risk of Bias tool for randomized trials (RoB 2) [22]. This increases the confidence in the internal validity of their findings. However, the presence of “some concerns” in one trial highlights the importance of protocol registration and transparent outcome reporting. In contrast, all non-randomized studies showed serious or critical risk of bias when assessed using Risk of Bias in Non-randomized Studies – of Interventions, Version 2 assesment tool (ROBINS-1-V2) [23]. Most concerning was the consistent presence of serious confounding, which is especially problematic in studies lacking statistical adjustment or using historical controls. Additionally, deficiencies in the classification of interventions and the measurement of outcomes contributed to further bias risk. These issues limit the interpretability of the non-randomized evidence and should temper any conclusions drawn from these data.

In future research, efforts should be made to reduce bias by using robust prospective designs, employing adequate control groups, and clearly defining interventions and outcome measures. Only through such high-quality clinical trials can we bridge the gap between promising basic science and effective clinical application in musculoskeletal medicine.

### Limitations

Limitations of this systematic review include the considerable variability in the HBOT protocols among the included studies, ranging from 1 to over 130 sessions and pressures between 1.3 and 2.5 ATA. Many studies lacked blinding or long-term follow-up. Particularly in conditions such as AVN, short treatment durations may fail to reflect the requirements for chronic remodeling. Moreover, outcome measures were inconsistent across studies, with few assessing functional return to sport or work.

Another limitation that needs to be mentioned is the lack of the protocol being registered in PROSPERO and a predefined protocol, which may theoretically have introduced risks of selective inclusion or post hoc interpretation of findings. However, all eligibility criteria are explicitly defined in the methods section, and all outcomes including null and negative findings are transparently reported, mitigating these concerns. No meta-analysis was planned and performed, due to the lack of homogenous data and no formal grey literature search was conducted. The narratively summarized review is more comparable to an evidence synthesis rather than independent prospective research. Future updates of this review need to be prospectively registered.

Two included studies reported incomplete protocol details: Currie et al. (2024) did not report HBOT pressure, session duration, or control group characteristics [36], and Ozturan et al. (2023) did not report session duration [35]. As no quantitative pooling was performed, these omissions did not affect the synthesis but limit the comparability of these studies with the remaining evidence.

The predominance of male athletes in muscle and ligament studies and the mixed or unreported sex distribution in bone studies limits the generalizability of findings across sexes.

Another limitation is the search restricted to PubMed and LIVIVO. While LIVIVO aggregates content from MEDLINE and additional life science databases, the omission of other big databases represents a limitation that may have resulted in missed studies, particularly given the sports medicine focus of this review. Future updates should incorporate these databases like Embase or SPORTDiscuss.

Publication bias cannot be excluded. The consistently positive findings across AVN and BME studies, all of which were observational in design, may overestimate the true treatment effect if negative or null results in these conditions remain unpublished.

## Conclusion

Hyperbaric oxygen therapy (HBOT) demonstrates promising therapeutic potential for selected musculoskeletal conditions, particularly early-stage avascular necrosis, bone marrow edema, and ligament injuries. However, clinical evidence remains heterogeneous, and its optimal role in musculoskeletal medicine is yet to be clearly defined. To better understand and make use of its regenerative capacity, future studies must focus on standardized treatment protocols, robust trial designs, and long-term functional outcomes, as well as exploring possible synergies with established and emerging regenerative interventions.

## Supplementary Information

Supplementary Material 1.

## Data Availability

All data generated or analyzed during this study are included in this published article.

## Abbreviations

ACL: Anterior cruciate ligament
AON: Aseptic osteonecrosis
AR: Anatomic region
ATA: Atmosphere Absolute
AVN: Avascular necrosis
AVNFH: Avascular Necrosis of Femoral Head
BME: Bone marrow edema
CD: Core decompression
CEBM: Oxford Centre for Evidence-Based Medicine
CG: Control group
CK: Creatine kinase
CO: Carbon monoxide
CSA: Cross-sectional area
DOMS: Delayed onset muscle soreness
EIMD: Exercise-induced muscle damage
G: Group
HBOT: Hyperbaric oxygen therapy
HIF-1α: Hypoxia-inducible factor
HMGB1: High mobility group box 1
IL-6: Interleukin-6
LDH: Lactate dehydrogenase
LOE: Muscle
MCL: Medial collateral ligament
max.: Maximum
mHHS: Modified Harris Hip Score
miR-107: MicroRNA-107
MRI: Magnetic resonance imaging
N: Number
NIRS: Near-infrared spectroscopy
NSAID: Non steroidal anti-inflammatory drugs
OKS: Oxford Knee Score
Ox: Oxygen
PHD2: Prolyl Hydroxylase Domain Protein 2
PRISMA: Preferred Reporting Items for Systematic Reviews and Meta-Analyses
PRP: Platelet-Rich Plasma
RAGE: Receptor for advanced glycation end products
RCT: Randomized controlled trial
RoB 2: Risk of Bias tool for randomized trials
ROBINS-I V2: The Risk of Bias in Non-randomized Studies – of Interventions, Version 2
RUNX2: Runt-related transcription factor 2
SC: Study concept
SF-12: Short Form 12 Dimensions
sign.: Significant
StO2: Oxygen saturation
THb: Blood volume
VAS: Visual analogue scale
VEGF: Vascular endothelial growth factor
VEGFA: Vascular endothelial growth factor A
WOMAC: Western Ontario and McMaster Universities Arthritis Index
